# Patient‐specific calibration of cone‐beam computed tomography data sets for radiotherapy dose calculations and treatment plan assessment

**DOI:** 10.1002/acm2.12293

**Published:** 2018-02-26

**Authors:** Michael MacFarlane, Daniel Wong, Douglas A. Hoover, Eugene Wong, Carol Johnson, Jerry J. Battista, Jeff Z. Chen

**Affiliations:** ^1^ London Regional Cancer Program London Health Science Center London ON Canada; ^2^ Department of Medical Biophysics Western University London ON Canada

**Keywords:** CBCT, DIR, dose tracking, treatment plan assessment

## Abstract

**Purpose:**

In this work, we propose a new method of calibrating cone beam computed tomography (CBCT) data sets for radiotherapy dose calculation and plan assessment. The motivation for this patient‐specific calibration (PSC) method is to develop an efficient, robust, and accurate CBCT calibration process that is less susceptible to deformable image registration (DIR) errors.

**Methods:**

Instead of mapping the CT numbers voxel‐by‐voxel with traditional DIR calibration methods, the PSC methods generates correlation plots between deformably registered planning CT and CBCT voxel values, *for each image slice*. A linear calibration curve specific to each slice is then obtained by least‐squares fitting, and applied to the CBCT slice's voxel values. This allows each CBCT slice to be corrected using DIR without altering the patient geometry through regional DIR errors.

A retrospective study was performed on 15 head‐and‐neck cancer patients, each having routine CBCTs and a middle‐of‐treatment re‐planning CT (reCT). The original treatment plan was re‐calculated on the patient's reCT image set (serving as the gold standard) as well as the image sets produced by voxel‐to‐voxel DIR, density‐overriding, and the new PSC calibration methods. Dose accuracy of each calibration method was compared to the reference reCT data set using common dose‐volume metrics and 3D gamma analysis. A phantom study was also performed to assess the accuracy of the DIR and PSC CBCT calibration methods compared with planning CT.

**Results:**

Compared with the gold standard using reCT, the average dose metric differences were ≤ 1.1% for all three methods (PSC: −0.3%; DIR: −0.7%; density‐override: −1.1%). The average gamma pass rates with thresholds 3%, 3 mm were also similar among the three techniques (PSC: 95.0%; DIR: 96.1%; density‐override: 94.4%).

**Conclusions:**

An automated patient‐specific calibration method was developed which yielded strong dosimetric agreement with the results obtained using a re‐planning CT for head‐and‐neck patients.

## 1 INTRODUCTION

Radiation treatments generally span several weeks and during this time, changes in patient weight, tumor volume and organ positioning can occur.^1^ These changes may substantially alter the radiation dose distribution within the patient, potentially resulting in degraded plan quality and suboptimal clinical outcomes.^2^

To ensure that a patient receives adequate treatment, a new re‐planning CT (reCT) data set may be acquired to dosimetrically assess plan quality and to evaluate whether treatment re‐planning has become necessary. Unfortunately, this workflow is often inefficient as it is difficult to distinguish *a priori* which patients require a reCT from those who do not. A promising solution is to use cone‐beam computed tomography (CBCT) image sets to dosimetrically assess plan quality, since these image sets are already routinely acquired prior to treatment for patient setup and monitoring. However, to perform dose calculations, accurate tissue density information must be extracted from the CBCT voxel values.

Normally, tissue density information is obtained through CT calibration curves, which are generated by scanning a plastic phantom containing various inserts of known electron density.^3^ For CBCT scans, the Hounsfield Units (HU) of an image set are highly dependent on many factors, including the size and material of the phantom, the materials placed in the phantom, and the imaging protocol used.^4^, ^5^, ^6^, ^7^ Furthermore, scattering conditions often differ between phantoms and patients when using a cone‐beam geometry. Due to this variability, HU‐to‐density calibration curves obtained with phantoms for CBCT lack sufficient robustness to be applicable to all patients and across all anatomical sites.^6^ Consequently, alternative methods of inferring tissue density have been proposed, such as: (a) population‐based calibration curves,^6^, ^7^ (b) multi‐level thresholding or bulk assignment of the HU or density values,^7^, ^8^, ^9^, ^10^ and (c) voxel‐to‐voxel mapping using deformable image registration (DIR).^10^, ^11^, ^12^

Although these CBCT calibration techniques have demonstrated some promising results, each method may have limitations in certain situations. For instance, population‐based calibration methods require unique calibration curves for each treatment site, and for each imaging protocol used. Bulk assignment techniques are dependent on the accuracy of automatic segmentation or thresholding of tissue regions, correct density assignments, or the time allotted to manually correct improperly delineated volumes. Similarly, DIR methods depend on the accuracy of the DIR algorithms, as regional DIR errors may significantly distort local anatomy and hence affect the density and dose evaluation within the region.^13^ This may be particularly problematic in sites such as the pelvis and thorax where large deformation errors frequently occur. Moreover, regional DIR errors could also alter the delineation of critical structures, thereby further affecting organ dose assessment and dose‐volume metrics.

To potentially resolve these limitations, we began development on an alternative patient‐specific CBCT calibration (PSC) technique that, while using DIR algorithms, is less sensitive to DIR uncertainties. Briefly, rather than mapping CT numbers voxel‐by‐voxel with DIR, we generate a systematic but patient‐specific calibration curve for each CBCT slice after registering CBCT to planning CT with DIR (see next section for details). This slice‐specific calibration curve is then applied to the CBCT slice to convert the voxel values to their “planning CT equivalent” values, without altering the patient geometry through regional DIR errors. Calibration curves are generated on a per‐slice basis since scattering conditions may vary axially and thereby affect the relationship between CBCT and planning CT HU values.

To evaluate whether this new PSC method improves dosimetric accuracy, we performed a retrospective patient study of 15 head‐and‐neck clinical cases, and a phantom study. The dosimetric accuracy of this PSC method was compared to a re‐planning CT (serving as the gold standard) and to other CBCT calibration methods proposed in literature (DIR mapping and bulk density assignment).

## 2 MATERIALS AND METHODS

### 2.A Patient selection

Fifteen head‐and‐neck cancer patients were selected at random from our institution database, all of whom had completed their treatment course and were referred for a reCT study at some point during their treatment course. This tumor site was selected due to the high frequency of treatment re‐planning. To minimize the dosimetric error resulting from anatomical differences, CBCTs acquired around the acquisition date of the reCT were reviewed and the CBCT with the most acceptable anatomical agreement with the reCT image set was selected. Patient and treatment related information are summarized in Table S1.

### 2.B Imaging

All CT and CBCT images were acquired as part of the patient's routine treatment course.

Original planning CT and re‐planning CT images were acquired on a Philips Brilliance Big Bore 16‐slice CT scanner (Philips Healthcare, Cleveland, OH). CT images were acquired with a full‐fan 120 kVp beam. The scanning parameters used to acquire each planning and re‐planning CT, can be found in Table S2 and S3, respectively. The CT images were reconstructed using the device's default filtered back‐projection algorithm, with a default slice thickness of 3 mm and slice size of 512 × 512. The voxel size varied between image sets as the CT operator would select the smallest field of view (FoV) required to cover the patient.^14^

CBCT images were acquired with either a Varian Truebeam or Clinac iX On‐Board Imaging (OBI) system (Varian Medical Systems, Palo Alto, CA). CBCT scans were acquired with either a standard (20 mA) or low‐dose (10 mA) protocol using a full‐fan 100 kVp beam with a full bow‐tie filter. The scanning parameters used to acquire each CBCT can be found in Table S4. CBCT scans were reconstructed by the treatment unit's OBI software (v 2.0‐2.1) which uses a Feldkamp‐Davis‐Kress (FDK) reconstruction algorithm with a Ram‐Lak filter.^15^, ^16^ Image slices were 384 × 384 in size when acquired with the Clinac iX's system, and 512 × 512 when acquired with the Truebeam's system.

### 2.C Creation of calibrated CBCT image sets

**Figure 1:**
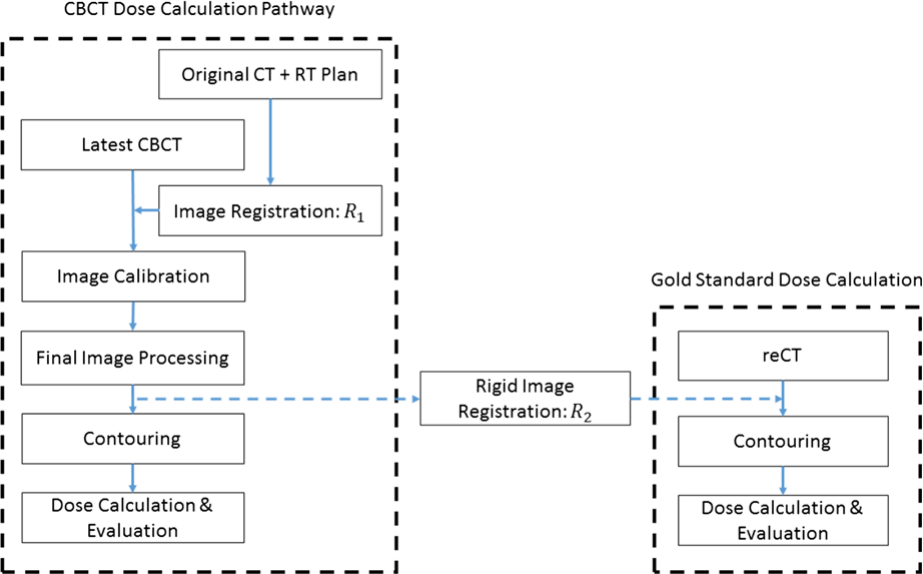
Schematic of the process used to generate a calibrated CBCT data set for dose calculation (left). The gold standard reCT data set is rigidly registered with the final calibrated CBCT data set for comparison (right).

Figure 1 outlines the general steps performed for each calibration method in this study. Details specific to each method will be described below.

### 2.D Patient‐specific calibration (PSC) method

The CBCT image sets were imported into a research version of the Pinnacle treatment planning system (v9.7, Philips Healthcare, Fitchburg, WI) along with the patient's original treatment planning CT data set (containing the CT scan, treatment plan, contours, and points of interest). The CBCT image set was first rigidly registered with the planning CT image set. The planning CT image set was then *deformably* registered to the CBCT image set using a fast‐symmetric Demon's algorithm implemented in Pinnacle, resulting in a deformed planning CT image set that was registered with the CBCT image set.^17^ The resulting deformed planning CT and the CBCT image sets were exported to Matlab (v2015a, MathWorks Inc, Natick, MA) for the patient‐specific calibration.

**Figure 2:**
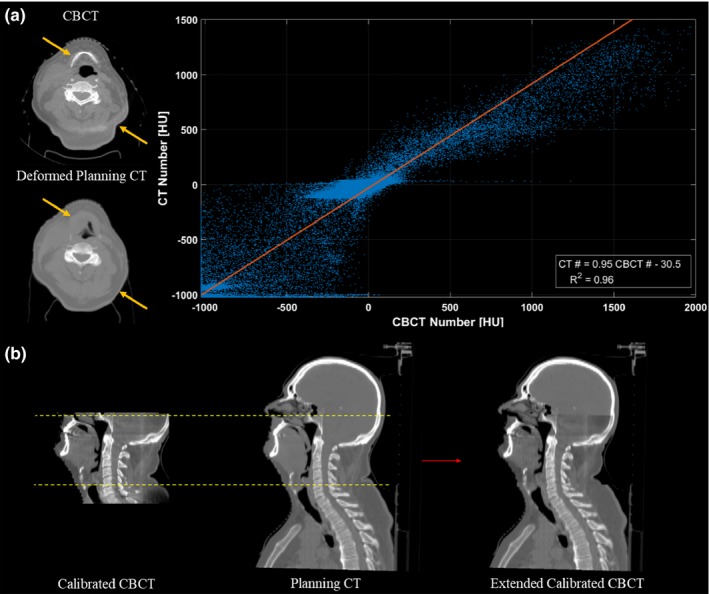
Illustration of the patient‐specific calibration (PSC) method. (a) An HU correlation plot is generated for each slice, between corresponding voxels of the CBCT and the deformed planning CT. Despite the presence of DIR errors (highlighted by the arrows), a strong slice‐specific linear calibration curve of the CBCT HU values to the planning CT HU values, can be obtained by least square fitting. (b) Once the linear mappings are applied, the calibrated CBCT image set is then rigidly registered, resampled and merged with the original planning CT image set to extend its field‐of‐view. Slices with poor correlation between the CBCT and the deformed planning CT voxel values (*R*_2_ < 0.8; outside of dashed lines) were replaced by the original planning CT.

A correlation plot of the voxel values was then generated for each slice between the deformed planning CT and CBCT image sets [Fig. 2(a)]. While deformation errors may have affected the correlation of HU values for some voxel pairs [such as those highlight by the arrows in Fig. 2(a)], most voxels within the slice will have been properly mapped by the DIR algorithm to planning CT HU values. Therefore, a strong relationship between the planning CT and CBCT HU values could be regressed from these correlation plots. With this relationship, we could scale the CBCT HU values to their CT‐equivalent values without introducing the regional DIR errors.

Linear calibration curve specific to each slice were obtained by least‐squares fitting of the correlation plots, and applied to each slice of the CBCT data set. These calibration curves were slice‐specific since scatter conditions will vary between slices of the CBCT, and therefore the relationship between CBCT and planning CT HU values (the model parameters regressed) may change.

As a final image processing step, the calibrated CBCT images were merged with the original planning CT images to extend the FoV, as shown in Fig. 2(b). Before merging, the calibrated CBCT images were rigidly registered with the original planning CT [using *R*_1_ in Fig. 1] and resampled with a linear interpolation algorithm so that the resolution of the CBCT matched that of the planning CT. Regions that were outside of the calibrated CBCT FoV or truncated during reconstruction were substituted with voxel values from the original planning CT images. Slices on the superior/inferior border with poor correlation between the voxel values of the CBCT and the deformed planning CT image set (*R* ^2^ < 0.8) were also replaced by the planning CT images. This usually occurred in the shoulder region where the CBCT FoV was insufficient to cover the whole patient, resulting in large deformation errors. By removing these slices, we could improve the anatomical matching at the junction of the CBCT and the original planning CT image set.

### 2.E Voxel‐to‐voxel DIR method

For the DIR method, the deformable image registration proceeded exactly as it did for the PSC method. Provided there are no significant DIR errors, a deformed planning CT will match the target CBCT while containing HU values from the source planning CT. Therefore, the deformed planning CT data set can be directly used to calculate the dose received at the time of treatment. After DIR, the deformed planning CT image set has the same dimensions and coordinates as the CBCT image set. Therefore, the deformed images were also rigidly registered, resampled, and merged with the original (undeformed) planning CT images to extend the field‐of‐view. Like the PSC method, the same slices on the superior/inferior border with poor correlation between the voxel values of the CBCT and the deformed planning CT (*R* ^2^ < 0.8) were replaced by the planning CT slices.

### 2.F Density‐override method

**Figure 3:**
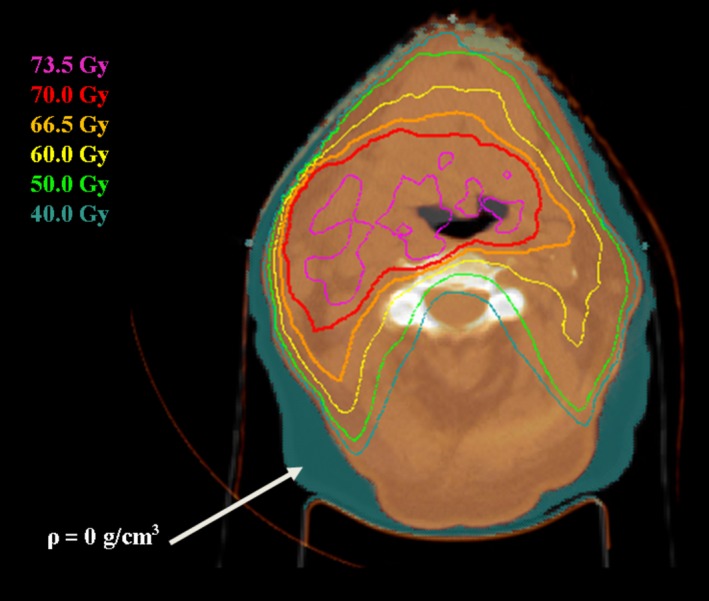
Illustration of the density‐override method. Regions of weight loss (shown in a teal colourwash) were assigned a density of 0 g/cm^3^ for dose calculations.

In the density‐override method, the CBCT image set was first rigidly registered with the original planning CT image set. Regions where soft tissue had become air (e.g., weight loss) or where air had been replaced by soft tissue (e.g., closed air cavity) were manually delineated on the original planning CT image set and assigned either water or air equivalent densities, accordingly. With these modifications, the major anatomical changes can be accounted for on the planning CT dataset, while continuing to use the original planning CT's HU values for dose calculations. This technique is similar to the algorithm proposed by van Zijtveld et al.^8^ and is illustrated in Fig. 3.

### 2.G Gold standard (reCT) for dose evaluation

The CBCT image sets calibrated by each method were imported back into the research version of Pinnacle, along with the patient's re‐planning CT data set (including contours). Each of the image sets were rigidly registered with the reCT image set (*R*_2_ in Fig. 1), taking care to match the original plan isocenter to the same anatomical location. The original treatment plan was then transferred to the reCT data set and dose was recomputed while maintaining the original beam layout and monitor units per beam. Dose was calculated on each data set using Pinnacle's Adaptive Collapsed Cone Convolution algorithm with inhomogeneity corrections.^18^ The dose grid was set to cover the entire patient CT images with 3 mm resolution in all directions. To minimize dosimetric errors, regions with metal streaking artifacts were delineated on each image set and assigned tissue equivalent densities for dose computation. Dose‐volume metrics evaluating tumor volume coverage and organ‐at‐risk (OAR) exposure were tabulated and served as the gold standard results.

### 2.H Contouring and dose metrics

The contours from the reCT data set were rigidly copied onto each calibrated CBCT image set based on the rigid registration *R*_2_. If necessary, these contours were manually adjusted to match the patient anatomy as seen on the calibrated image set. Dose metrics evaluating tumor volume coverage and organ‐at‐risk (OAR) exposure were again tabulated and compared to the gold standard results.

### 2.I Gamma analysis

Dose distributions computed on each of the calibrated image sets were compared to the gold standard dose distribution using the SlicerRT extension (v 0.18.0) of 3D Slicer (v 4.6.2).^19^, ^20^ A 3D gamma analysis was restricted to a region inside the original CBCT volume and excluded voxels within 3 mm of the surface so that uncertainties in surface dose were omitted. The analysis was performed with a low‐dose threshold of 10% (relative to the maximum point dose on the reCT data set), and acceptance criteria of 3% dose‐difference and 3 mm distance‐to‐agreement. The gamma pass rate (percentage of voxels with γ < 1) was tabulated.

### 2.J Statistical analysis

A one‐way repeated measures MANOVA was performed in the Statistical Package for the Social Sciences (SPSS v23, IBM Corp, Chicago, IL) to assess whether the image set used for dose calculations influenced the collective dose metric values. Univariate analysis followed when the MANOVA test was significant, along with *post‐hoc* pair‐wise Student's *t*‐tests when appropriate. A one‐way repeated measure ANOVA was also performed to find statistical differences between the gamma pass rates. A 5% threshold for statistical significance (*P* = 0.05) was used.

### 2.K Phantom study

A phantom study was also performed to assess the accuracy of the DIR and PSC CBCT calibration methods. A planning CT and CBCT (Clinac iX) scan were acquired of the CIRS 062 inner “head” phantom with various material inserts (Computerized Imaging Reference Systems Inc, Norfolk, VA).

**Figure 4:**
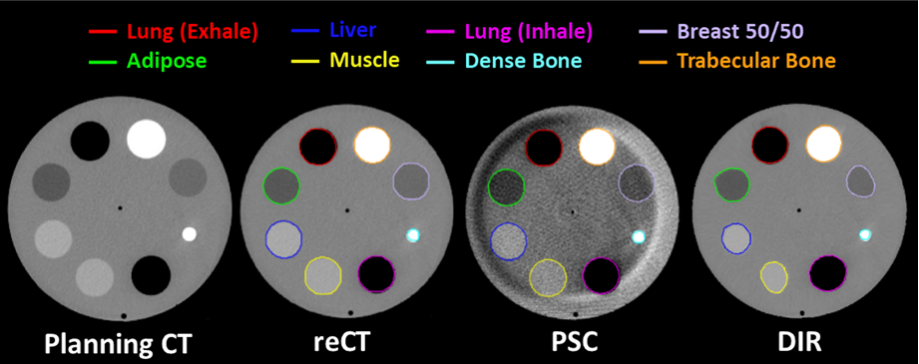
The image sets and contours produced for the phantom study. A simulated reCT was produced by reducing the Planning CT's in‐plane voxel size by 5%. The CBCT voxel size was also adjusted by 5%. The CBCT was then calibrated using both the PSC and DIR methods. The inserts were delineated on each image and the average density and Dice Coefficient (relative to the reCT) was computed for comparison.

To simulate weight loss with the phantom, a simulated reCT image set was created by reducing the planning CT's in‐plane dimensions by 5% (yielding an equivalent depth reduction of 4.5 mm) as shown in Fig. 4. Similarly, the in‐plane dimensions of the CBCT images were reduced by 5% to match the simulated reCT.

The original planning CT, simulated reCT and CBCT image sets were imported into the research version of Pinnacle. The CBCT was then calibrated using both the DIR and the PSC CBCT calibration methods described above. Merging of the calibrated CBCT image sets with the original planning CT was not required as the CBCT FoV was sufficient to capture the entire phantom. The calibrated CBCT images were then rigidly registered with the reCT image set. The inserts in the phantom were manually delineated on each image set (as shown in Fig. 4) and the average density and Sørensen‐Dice similarity coefficient (compared to the reCT contour) were calculated for each insert and each image set.

## 3 RESULTS

**Figure 5:**
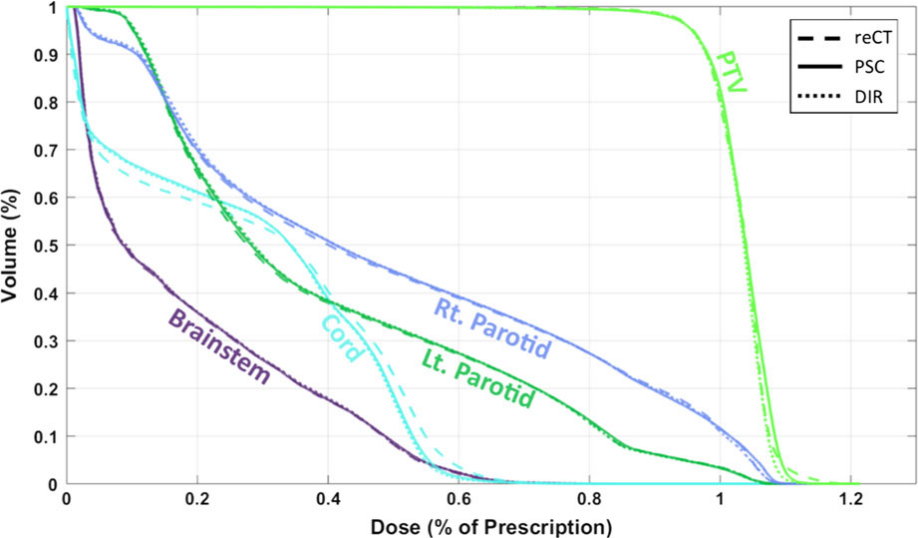
Average dose‐volume histograms of fifteen plans, calculated with the gold‐standard reCT image set (dashed line), and the CBCT calibrated with the patient‐specific calibrated method (PSC, solid line), and the DIR method (DIR, dotted line).

**Figure 6:**
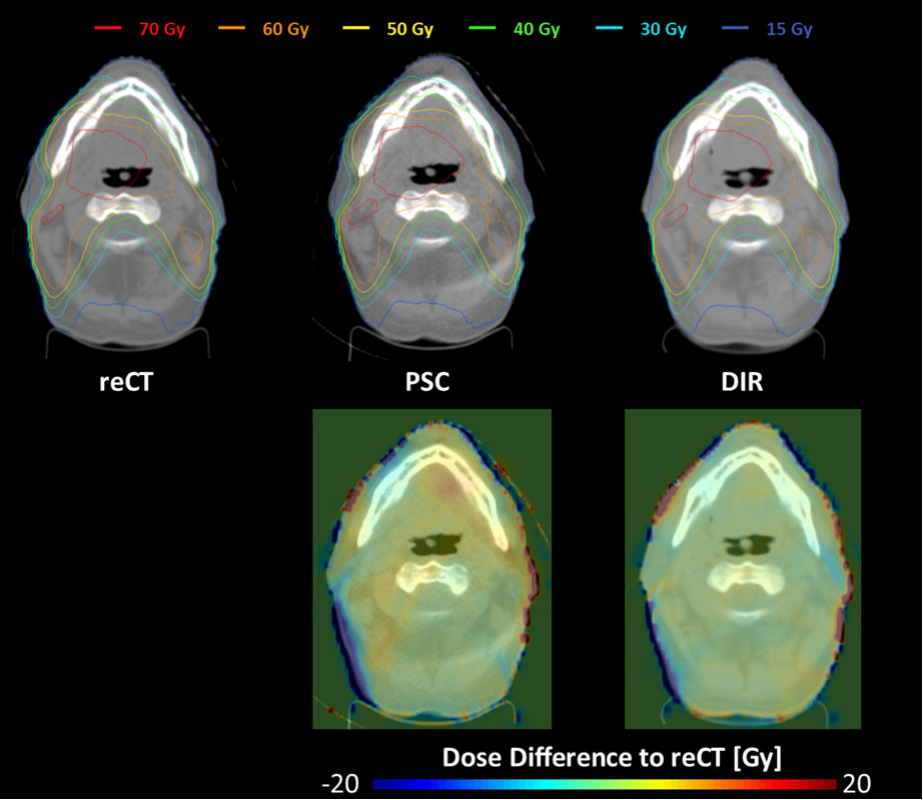
(Upper) Sample dose distributions from patient 1 for the plans calculated on the gold standard reCT (left), PSC calibrated CBCT (middle), and DIR calibrated CBCT (right) image sets. (Lower) Differences between the PSC, DIR calculated dose distribution and the reCT calculated dose distribution.

Table 1 shows the difference of various dose metrics compared to the gold standard values, averaged over all patients and normalized to the prescription dose (due to different prescription doses between patients). Figures 5 and 6 show the average dose‐volume histogram of the 15 patients and a sample patient dose distribution, respectively.

**Table 1:** Mean (standard deviation) dose metric differences compared to the gold standard reCT, normalized by the prescribed dose. Dose metrics that were significantly different to the reCT are indicated with the *(*P* < 0.05) and ^†^(*P* < 0.01)

ROI	Dose metric	PSC method (%)	DIR method (%)	Density‐override (%)
PTV	D_95%_	−1.1 (1.0)	−0.9 (1.0)	−0.8 (2.8)
	Mean	−0.5 (0.8)	−1.0 (0.8)^†^	−1.5 (0.8)^†^
	D_2%_	0.0 (1.3)	−0.8 (1.2)^†^	−1.5 (1.3)^†^
Brainstem	D_0.1 cc_	0.6 (1.0)	0.0 (1.2)	−0.5 (1.5)
Cord	D_0.1 cc_	−2.0 (2.5)*	−3.0 (3.3)^†^	−3.4 (3.1)^†^
Lt. parotid	Mean	0.7 (1.5)	0.3 (2.4)	0.5 (1.9)
Rt. parotid	Mean	0.5 (2.2)	0.2 (3.1)	−0.4 (2.4)

ROI, region of interest; PSC, patient‐specific calibration; DIR, deformable image registration; D_XX_, minimum dose to the most irradiated XX volume, specified in percent or cubic centimeters (cc), as indicated.

On average, dose metric differences were ≤ 1.1% for all three methods, with the PSC method providing marginally better agreement (−0.3 ± 1.0%, mean ± standard deviation) compared to the DIR (−0.7 ± 1.1%) and density‐override (−1.1 ± 1.2%) methods. Multivariate testing revealed that the image set used for dose calculation had a statistically significant effect on the dose metric values (*P* < 0.001). Further univariate analysis and pair‐wise *t*‐testing found that the spinal cord maximum dose D_0.1 cc_ metric was systematically underestimated by all three methods (*P* < 0.05). Furthermore, both DIR and density‐override methods also systematically underestimated both the PTV mean dose metric (*P* < 0.01) and D_02_ metric (*P* < 0.01), whereas the PSC method did not. The level of statistical significance of the Student's *t*‐test is indicated by asterisks (*P* < 0.05) and daggers (*P* < 0.01) in Table 1.

The results of the 3D gamma analysis were found to be similar across all three techniques (*P* = 0.41), with the average (standard deviation) gamma pass rates of 95.0% (3.0%), 96.1% (3.3%), and 94.4% (4.4%) for the PSC, DIR, and density‐override methods, respectively.

Results from the CIRS phantom study are provided in Table 2. Relative to the reCT scan, the DIR calibrated CBCT provided very similar densities for every insert in the phantom. However, significant distortions were introduced into the image set as a result of the DIR errors, as evident by the Dice coefficient values and by visual inspection of the deformed CT in Fig. 4. Conversely, the PSC calibrated CBCT provides better anatomy matching than DIR, with higher Dice coefficients. Despite the presence of crescent artifacts, the PSC method also improves the average density accuracy for most materials, relative to the uncalibrated CBCT, as shown in Table 2.

**Table 2:** The average density and Sørensen‐Dice similarity coefficient (compared to the reCT) calculated for each insert in the CIRS 062 phantom, and for each image set

Material	Computed density (g/cm^3^)			Dice coefficient	
[True density (g/cm^3^)]	DIR	PSC	CBCT	PSC	DIR
Exhaled lung (0.52)	0.53	0.52	0.47	0.98	0.96
Adipose (0.93)	0.93	0.90	0.87	0.99	0.94
Liver (1.05)	1.05	1.04	1.02	0.96	0.77
Muscle (1.05)	1.05	1.04	1.00	0.97	0.73
Inhaled lung (0.24)	0.26	0.27	0.21	0.97	0.97
Dense bone (1.55)	1.55	1.58	1.57	0.86	0.80
Breast 50/50 (0.96)	0.96	0.95	0.91	0.96	0.79
Trabecular bone (1.20)	1.19	1.21	1.20	0.94	0.92

On average, it took about 30 min to perform the full DIR and PSC calibration workflow, with the bulk of the time spent on dose calculations and transferring the image sets between systems for merging and/or calibration. Of those 30 min, under a minute was spent running the DIR, and only a few seconds were spent calibrating and merging the CBCT with planning CT image set using the PSC method. The density‐override techniques took longer (~50 min) because the contours delineating anatomical changes were generated manually.

## 4 DISCUSSION

We have developed a patient‐specific method of calibrating CBCTs for dose tracking and plan assessment, and compared it with other methods for the head‐and‐neck site.^8^, ^11^ The results show that slightly better dosimetric agreement with the gold standard reCT can be obtained when using this patient‐specific calibration (PSC) method, although each method demonstrated sufficient accuracy for plan re‐assessment during radiotherapy.

It is worth noting that the spinal cord dose was poorly estimated by all three methods (D_0.1 cc_ in Table 1, Fig. 5). This was caused by a few select patients who had slight variations of the spinal cord positioning in regions of steep dose gradients (due to differences in setup between the reCT and the CBCT studies). It should also be noted that the gamma pass rates presented in this study are lower than other published results. For example, both van Zijtveld et al.^8^ and Veiga et al.^11^ reported similar gamma pass rates for the head‐and‐neck site when using a stricter 2%, 2 mm acceptance criteria. The difference in gamma pass rates could be attributed to differences in the study design. For instance, Veiga et al.^11^ performed their DIR method on simulated CBCTs, which were created by deforming the selected CBCT to match the reCT.

The results from this study illustrate the pros and cons of the three CBCT calibration methods. While the density override method is easy to implement on available treatment planning systems, it cannot account for internal anatomical changes and it can be very time consuming to perform. Furthermore, the observed dosimetric accuracy of this technique is not as high as the other CBCT calibration techniques (Table 1). The DIR method is less sensitive to the CBCT artifacts (such as the crescent artifact visible in Fig. 4) and provides sufficiently accurate tissue density and dosimetric information (Table 2, Fig. 5). However, DIR methods may introduce distortions into the image through DIR errors [Figs. 2(a), 4 and Table 2) that can affect OAR delineations and their dosimetric evaluations (Table 1). On the other hand, the PSC method is less sensitive to regional DIR errors as it maintains the patient anatomy from the CBCT, resulting in higher Dice similarity coefficients as shown in Table 2. While the PSC method preserves the patient anatomy, it also preserves the noise and any artifacts present in the CBCT images (Fig. 4). It also produces slightly less accurate densities than the DIR methods (Table 2). Neither of these limitations appeared to have considerable influence on the dosimetric performance of the PSC method (Table 1, Fig. 5).

While distortions introduced by DIR calibration did not have substantial influence on the dosimetric accuracy in the head‐and‐neck site studied here (Table 1, Figs. 5 and 6), the same may not be true in sites such as pelvis or thorax where large DIR distortions are commonplace at tissue‐air interfaces, such as the bowel. Therefore, the PSC method could be potentially advantageous for these sites and will be investigated in the future.

Based on Table 2, the PSC method improves the average density accuracy of the uncalibrated CBCT, for most materials inserted in the phantom. However, since there were relatively low amounts of high‐density (bone) material in each slice, the calibration curves used by the PSC method were primarily fitted for lower density materials and not higher density materials. As a result, the density of higher density materials were not corrected by the PSC method. A future version of this PSC method could potentially be improved using a piece‐wise continuous linear calibration curves that calibrates both lower and higher density materials separately. Furthermore, the limited FoV, noise, and artifacts present in CBCTs may pose additional challenges in sites such as the pelvis or thorax.^21^, ^22^ Therefore, more sophisticated methods of extending the CBCT field‐of‐view (such as fusion‐aligned reprojection techniques^23^), and reducing the noise and artifacts present in the CBCT, will be investigated in the future. The performance of this method will also need to be verified on other CBCT imaging systems, and in other treatment sites.

**Figure 7:**
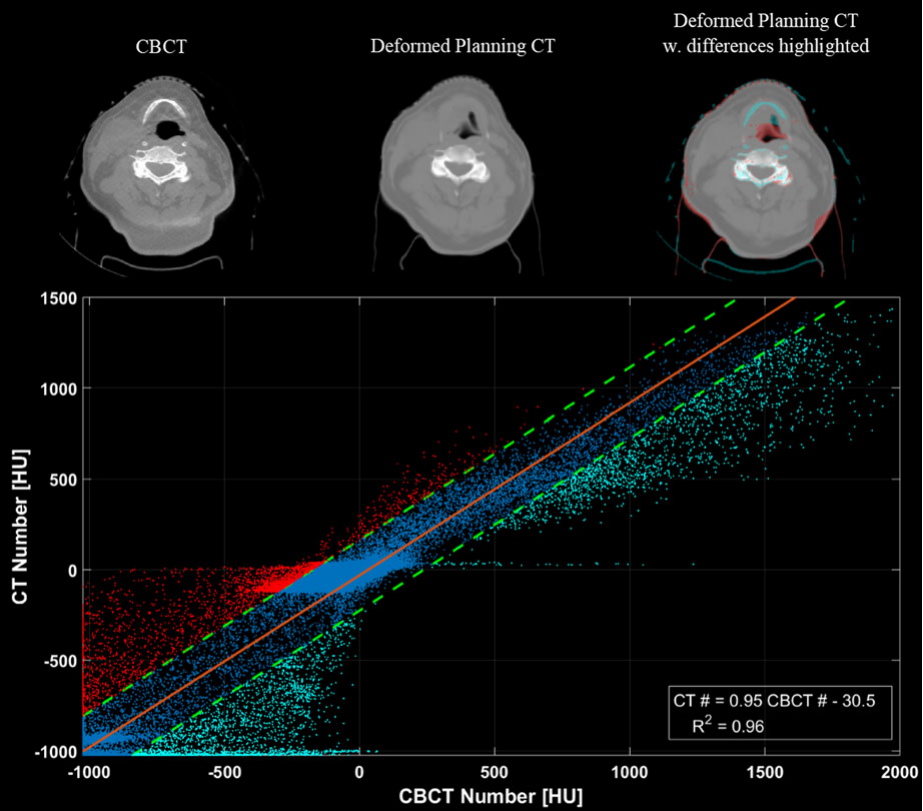
Illustration of how the linear calibration tool may be used to highlight regions of deformation error. The bottom frame shows the correlation plot generated for this slice. The linear mapping used to calibrate the slice is shown in orange, while the upper and lower bounds of the 95% confidence interval (CI) are shown as the dashed green lines. Data‐points falling outside of the 95% CI are labelled in red and blue on the plot, and are also highlighted on the top‐right deformed planning CT to show regions where the CBCT (top‐left) and deformed planning CT (top‐middle) differ due to DIR errors.

Finally, in addition to calibrating CBCT for dose calculations, the calibration curves used in the PSC method can also be used to quickly identify regions of potential DIR error on a deformed CT. For example, if one highlights the voxels outside of the 95% confidence interval of the calibration curve, regions where the CBCT and the deformed CT differed substantially can be easily visualized. An example of this application is provided in Fig. 7.

## 5 CONCLUSION

A patient‐specific CBCT calibration method has been proposed and tested for the head‐and‐neck site. Compared to a gold standard reCT dose distribution, average differences in dose metric values were ≤ 1.1% for all calibration methods tested, although the PSC method showed slightly better performance. Some advantages of the PSC method are that it preserves the patient anatomy, accounts for variable scattering per CT slice, can be uniquely applied to each patient, is computationally efficient, and may also be used to display errors introduced by DIR algorithms.

## CONFLICTS OF INTEREST

This work received partial funding by Philips Healthcare.

## Supporting information


**Table S1.** Summary of Patient Treatment Information.
**Table S2.** Summary of the planning CT acquisition.
**Table S3.** Summary of the re‐planning CT acquisition.
**Table S4.** Summary of the CBCT acquisition.

## References

[1] Barker Jr. JL , Garden AS , Ang KK , et al. Quantification of volumetric and geometric changes occurring during fractionated radiotherapy for head‐and‐neck cancer using an integrated CT/linear accelerator system. Int J Radiat Oncol Biol Phys. 2004;59:960–970. 15234029 10.1016/j.ijrobp.2003.12.024

[2] Hansen EK , Bucci MK , Quivey JM , Weinberg V , Xia P . Repeat CT imaging and replanning during the course of IMRT for head‐and‐neck cancer. Int J Radiat Oncol Biol Phys. 2006;64:355–362. 16256277 10.1016/j.ijrobp.2005.07.957

[3] Saw CB , Loper A , Komanduri K , Combine T , Huq S , Scicutella C . Determination of CT‐to‐density conversion relationship for image‐based treatment planning systems. Med Dosim. 2005;30:145–148. 16112465 10.1016/j.meddos.2005.05.001

[4] Yoo S , Yin FF . Dosimetric feasibility of cone‐beam CT‐based treatment planning compared to CT‐based treatment planning. Int J Radiat Oncol Biol Phys. 2006;66:1553–1561. 17056197 10.1016/j.ijrobp.2006.08.031

[5] Hatton J , McCurdy B , Greer PB . Cone beam computerized tomography: the effect of calibration of the Hounsfield unit number to electron density on dose calculation accuracy for adaptive radiation therapy. Phys Med Biol. 2009;54:N329–N346. 19590116 10.1088/0031-9155/54/15/N01

[6] Richter A , Hu Q , Steglich D , et al. Investigation of the usability of conebeam CT data sets for dose calculation. Rad Oncol. 2008;3:42. 10.1186/1748-717X-3-42 PMC2648965 19087250

[7] Fotina I , Hopfgartner J , Stock M , Steininger T , Lütgendorf‐Caucig C , Georg D . Feasibility of CBCT‐based dose calculation: comparative analysis of HU adjustment techniques. Radiother Oncol. 2012;104:249–256. 22809588 10.1016/j.radonc.2012.06.007

[8] van Zijtveld M , Dirkx M , Heijmen B . Correction of conebeam CT values using a planning CT for derivation of the ‘‘dose of the day’’. Radiother Oncol. 2007;85:195–200. 17936387 10.1016/j.radonc.2007.08.010

[9] Chen S , Le Q , Mutaf Y , et al. Feasibility of CBCT‐based dose with a patient‐specific stepwise HU‐to‐density curve to determine time of replanning. J Appl Clin Med Phys. 2017;18:64–69. 28703475 10.1002/acm2.12127 PMC5875829

[10] Onozato Y , Kadoya N , Fujita Y , et al. Evaluation of on‐board kV cone beam computed tomography – based dose calculation with deformable image registration using Hounsfield unit modifications. Int J Radiat Oncol Biol Phys. 2014;89:417–423. 10.1016/j.ijrobp.2014.02.007 24685445

[11] Veiga C , McClelland J , Moinuddin S , et al. Toward adaptive radiotherapy for head and neck patients: feasibility study on using CT‐to‐CBCT deformable registration for “dose of the day” calculations. Med Phys. 2014;41:031703. 24593707 10.1118/1.4864240

[12] Moteabbed M , Sharp GC , Wang Y , Trofimov A , Efstathiou JA , Lu HM . Validation of a deformable image registration technique for cone beam CT‐based dose verification. Med Phys. 2014;42:196–205. 10.1118/1.4903292 PMC4277556 25563260

[13] Vickress J , Battista J , Barnett R , Yartsev S . Representing the dosimetric impact of deformable image registration errors. Phys Med Biol. 2017;62:N391–N403. 28800299 10.1088/1361-6560/aa8133

[14] Philips CT Clinical Science . Respiratory motion management for CT. Philips White Paper; 2013, Nr. 4522 962 91291.

[15] Han X , Pearson E , Pelizzari C , et al. Algorithm‐enabled exploration of image‐quality potential of cone‐beam CT in image‐guided radiation therapy. Phys Med Biol. 2015;60:4601–4633. 26020490 10.1088/0031-9155/60/12/4601 PMC4610380

[16] Ramachandran G , Lakshminarayanan A . Three‐dimensional reconstruction from radiographs and electron micrographs: application of convolutions instead of Fourier transforms. Proc Natl Acad Sci USA. 1971;68:2236–2240. 5289381 10.1073/pnas.68.9.2236 PMC389392

[17] Dru F , Vercauteren T . An ITK implementation of the symmetric log‐domain diffeomorphic demons algorithm. Insight J. 2009 https://hal.inria.fr/hal-00813744. Accessed February 15, 2018.

[18] McNutt T . Dose calculations – collapsed cone convolution and delta pixel beam. Pinnacle White Paper 2007, Nr. 4535 983 02474/870.

[19] Low DA , Harms WB , Mutic S , Purdy JA . A technique for the quantitative evaluation of dose distributions. Med Phys. 1998;25:656–661. 9608475 10.1118/1.598248

[20] Pinter C , Lasso A , Wang A , Jaffray D , Fichtinger G . SlicerRT: radiation therapy research toolkit for 3D Slicer. Med Phys. 2012;39:6332–6338. 23039669 10.1118/1.4754659

[21] Niu T , Sun M , Star‐Lack J , Gao H , Fan Q , Lei Z . Shading correction for on‐board cone‐beam CT in radiation therapy using planning MDCT images. Med Phys. 2010;37:5395–5406. 21089775 10.1118/1.3483260

[22] Marchant TE , Moore CJ , Rowbottom CG , MacKay RI , Williams PC . Shading correction algorithm for improvement of cone‐beam CT images in radiotherapy. Phys Med Biol. 2008;53:5719–5733. 18824785 10.1088/0031-9155/53/20/010

[23] Ruchala KJ , Olivera GH , Kapatoes JM , Reckwerdt PJ , Mackie TR . Methods for improving limited field‐of‐view radiotherapy reconstructions using imperfect a priori images. Med Phys. 2002;29:2590–2605. 12462726 10.1118/1.1513163

