# Supplementary material for: Patient‐specific calibration of cone‐beam computed tomography data sets for radiotherapy dose calculations and treatment plan assessment
Source: J Appl Clin Med Phys. 2018 Feb 26;19(2):249–57. doi: 10.1002/acm2.12293 (PMC5849848; doi:10.1002/acm2.12293)
Supplement: Supplementary file 1 — Table S1. Summary of Patient Treatment Information. Table S2. Summary of the planning CT acquisition. Table S3. Summary of the re‐planning CT acquisition. Table S4. Summary of the CBCT acquisition. [file ACM2-19-249-s001.doc]

| **Supplementary Table 1:** Summary of Patient Treatment Information | | | |  |
| --- | --- | --- | --- | --- |
| **No.** | **Primary Disease Site** | **Delivery Method** | **Re-planned After** | |
| 1 | Tongue | 2 Coplanar 360^o^ Arcs | 18/35 Fxns | |
| 2 | Mouth Floor | 2 Coplanar 360^o^ Arcs | 9/30 Fxns | |
| 3 | Right Neck | 2 Coplanar 360^o^ Arcs | 27/35 Fxns | |
| 4 | Tongue | 2 Coplanar 360^o^ Arcs | 29/35 Fxns | |
| 5 | Oropharynx | 2 Coplanar 360^o^ Arcs | 17/35 Fxns | |
| 6 | Mouth | 2 Coplanar 180^o^ Arcs | 12/30 Fxns | |
| 7 | Tonsils | 2 Coplanar 360^o^ Arcs | 21/35 Fxns | |
| 8 | Tonsils | 2 Coplanar 360^o^ Arcs | 17/35 Fxns | |
| 9 | Tongue | 2 Coplanar 360^o^ Arcs | 14/35 Fxns | |
| 10 | Tonsils | 2 Coplanar 360^o^ Arcs | 26/35 Fxns | |
| 11 | Tonsils | 2 Coplanar 360^o^ Arcs | 20/35 Fxns | |
| 12 | Tongue | 2 Coplanar 360^o^ Arcs | 27/35 Fxns | |
| 13 | Tongue | 2 Coplanar 360^o^ Arcs | 24/35 Fxns | |
| 14 | Neck | 2 Coplanar 360^o^ Arcs | 21/35 Fxns | |
| 15 | Nasal Cavity | 2 Non-coplanar Arcs | 17/30 Fxns | |
| Abbreviations: Fxns, fractions. | | | |  |

| **Supplementary Table 2:** Summary of the planning CT acquisition. | | | | | | | | |  |
| --- | --- | --- | --- | --- | --- | --- | --- | --- | --- |
| **No.** | **Device** | **Acquisition Date** | **Energy [kV]** | **X-Ray Current [mA]** | **Exposure Time [ms]** | **Exposure**  **[mAs]** | **Slices** | **Voxel Size [mm]** | |
| 1 | Brilliance Big Bore | 11/27/2012 | 120 | 283 | 1060 | 300 | 146 | 1.01 x 1.01 x 3 | |
| 2 | Brilliance Big Bore | 4/5/2016 | 120 | 245 | 1224 | 300 | 141 | 1.10 x 1.10 x 3 | |
| 3 | Brilliance Big Bore | 5/15/2015 | 120 | 281 | 1068 | 300 | 135 | 1.00 x 1.00 x 3 | |
| 4 | Brilliance Big Bore | 5/11/2016 | 120 | 242 | 1240 | 300 | 155 | 1.22 x 1.22 x 3 | |
| 5 | Brilliance Big Bore | 8/14/2013 | 120 | 283 | 1060 | 300 | 126 | 1.02 x 1.02 x 3 | |
| 6 | Brilliance Big Bore | 9/25/2013 | 120 | 281 | 1068 | 300 | 151 | 0.97 x 0.97 x 3 | |
| 7 | Brilliance Big Bore | 12/9/2013 | 120 | 244 | 1230 | 300 | 140 | 1.09 x 1.09 x 3 | |
| 8 | Brilliance Big Bore | 12/3/2013 | 120 | 281 | 1068 | 300 | 158 | 1.00 x 1.00 x 3 | |
| 9 | Brilliance Big Bore | 1/27/2014 | 120 | 281 | 1068 | 300 | 136 | 0.93 x 0.93 x 3 | |
| 10 | Brilliance Big Bore | 5/13/2014 | 120 | 283 | 1060 | 300 | 134 | 1.02 x 1.02 x 3 | |
| 11 | Brilliance Big Bore | 6/8/2015 | 120 | 244 | 1230 | 300 | 120 | 1.18 x 1.18 x 3 | |
| 12 | Brilliance Big Bore | 6/24/2015 | 120 | 208 | 1447 | 301 | 135 | 1.30 x 1.30 x 3 | |
| 13 | Brilliance Big Bore | 4/11/2016 | 120 | 281 | 1068 | 300 | 140 | 0.93 x 0.93 x 3 | |
| 14 | Brilliance Big Bore | 4/11/2016 | 120 | 283 | 1060 | 300 | 121 | 0.93 x 0.93 x 3 | |
| 15 | Brilliance Big Bore | 9/23/2015 | 120 | 244 | 1230 | 300 | 124 | 1.05 x 1.05 x 3 | |

| **Supplementary Table 3:** Summary of the re-planning CT acquisition. | | | | | | | | |  |
| --- | --- | --- | --- | --- | --- | --- | --- | --- | --- |
| **No.** | **Device** | **Acquisition Date** | **Energy [kV]** | **X-Ray Current [mA]** | **Exposure Time [ms]** | **Exposure**  **[mAs]** | **Slices** | **Voxel Size [mm]** | |
| 1 | Brilliance Big Bore | 1/8/2013 | 120 | 244 | 1230 | 300 | 153 | 1.20 x 1.20 x 3 | |
| 2 | Brilliance Big Bore | 5/26/2016 | 120 | 244 | 1230 | 300 | 137 | 1.08 x 1.08 x 3 | |
| 3 | Brilliance Big Bore | 7/8/2015 | 120 | 244 | 1230 | 300 | 167 | 1.13 x 1.13 x 3 | |
| 4 | Brilliance Big Bore | 6/29/2016 | 120 | 244 | 1230 | 300 | 131 | 1.10 x 1.10 x 3 | |
| 5 | Brilliance Big Bore | 9/19/2013 | 120 | 244 | 1230 | 300 | 158 | 1.13 x 1.13 x 3 | |
| 6 | Brilliance Big Bore | 10/21/2013 | 120 | 281 | 1068 | 300 | 144 | 1.04 x 1.04 x 3 | |
| 7 | Brilliance Big Bore | 1/28/2014 | 120 | 244 | 1230 | 300 | 156 | 1.19 x 1.19 x 3 | |
| 8 | Brilliance Big Bore | 1/10/2014 | 120 | 281 | 1068 | 300 | 153 | 0.98 x 0.98 x 3 | |
| 9 | Brilliance Big Bore | 2/28/2014 | 120 | 244 | 1230 | 300 | 137 | 1.16 x 1.16 x 3 | |
| 10 | Brilliance Big Bore | 6/27/2014 | 120 | 281 | 1068 | 300 | 134 | 1.03 x 1.03 x 3 | |
| 11 | Brilliance Big Bore | 7/24/2015 | 120 | 244 | 1230 | 300 | 146 | 1.05 x 1.05 x 3 | |
| 12 | Brilliance Big Bore | 8/11/2015 | 120 | 281 | 1068 | 300 | 137 | 0.97 x 0.97 x 3 | |
| 13 | Brilliance Big Bore | 5/26/2016 | 120 | 244 | 1230 | 300 | 143 | 1.15 x 1.15 x 3 | |
| 14 | Brilliance Big Bore | 5/10/2016 | 120 | 281 | 1068 | 300 | 145 | 1.03 x 1.03 x 3 | |
| 15 | Brilliance Big Bore | 11/3/2015 | 120 | 245 | 1224 | 300 | 125 | 1.11 x 1.11 x 3 | |

| **Supplementary Table 4:** Summary of the CBCT acquisition. | | | | | | | | |
| --- | --- | --- | --- | --- | --- | --- | --- | --- |
| **No.** | **Device** | **Acquisition Date** | **Energy [kV]** | **X-Ray Current [mA]** | **Exposure Time [ms]** | **Exposure**  **[mAs]** | **Slices** | **Voxel Size [mm]** |
| 1 | Clinac iX | 1/8/2013 | 100 | 20 | 7000 | 140 | 70 | 0.65 x 0.65 x 2.5 |
| 2 | Truebeam | 5/26/2016 | 100 | 20 | 7450 | 149 | 93 | 0.51 x 0.51 x 2.0 |
| 3 | Truebeam | 7/15/2015 | 100 | 20 | 7450 | 149 | 93 | 0.51 x 0.51 x 2.0 |
| 4 | Truebeam | 7/5/2016 | 100 | 20 | 7500 | 150 | 93 | 0.51 x 0.51 x 2.0 |
| 5 | Clinac iX | 9/19/2013 | 100 | 20 | 7050 | 141 | 70 | 0.65 x 0.65 x 2.5 |
| 6 | Truebeam | 10/21/2013 | 100 | 20 | 7250 | 145 | 89 | 0.51 x 0.51 x 2.0 |
| 7 | Clinac iX | 1/28/2014 | 100 | 20 | 7000 | 140 | 70 | 0.65 x 0.65 x 2.5 |
| 8 | Truebeam | 1/10/2014 | 100 | 10 | 7200 | 72 | 70 | 0.49 x 0.49 x 2.5 |
| 9 | Clinac iX | 2/28/2014 | 100 | 10 | 7000 | 70 | 70 | 0.65 x 0.65 x 2.5 |
| 10 | Clinac iX | 6/26/2014 | 100 | 20 | 7250 | 145 | 70 | 0.65 x 0.65 x 2.5 |
| 11 | Clinac iX | 7/23/2015 | 100 | 20 | 6950 | 139 | 70 | 0.65 x 0.65 x 2.5 |
| 12 | Clinac iX | 8/11/2015 | 100 | 20 | 7200 | 144 | 70 | 0.65 x 0.65 x 2.5 |
| 13 | Clinac iX | 5/26/2016 | 100 | 10 | 7000 | 70 | 70 | 0.65 x 0.65 x 2.5 |
| 14 | Clinac iX | 5/10/2016 | 100 | 20 | 7200 | 144 | 70 | 0.65 x 0.65 x 2.5 |
| 15 | Clinac iX | 11/3/2015 | 100 | 20 | 7000 | 140 | 70 | 0.65 x 0.65 x 2.5 |
